# The double‐edged role of neutrophil heterogeneity in inflammatory diseases and cancers

**DOI:** 10.1002/mco2.325

**Published:** 2023-07-23

**Authors:** Wencheng Zhou, Xinran Cao, Qiang Xu, Jiao Qu, Yang Sun

**Affiliations:** ^1^ Department of Pharmacy The First Affiliated Hospital of Zhejiang Chinese Medical University (Zhejiang Provincial Hospital of Chinese Medicine) Hangzhou China; ^2^ State Key Laboratory of Pharmaceutical Biotechnology Department of Biotechnology and Pharmaceutical Sciences School of Life Science Nanjing University Nanjing China

**Keywords:** cancers, inflammatory diseases, neutrophil extracellular traps, neutrophil heterogeneity, single‐cell sequencing

## Abstract

Neutrophils are important immune cells act as the body's first line of defense against infection and respond to diverse inflammatory cues. Many studies have demonstrated that neutrophils display plasticity in inflammatory diseases and cancers. Clarifying the role of neutrophil heterogeneity in inflammatory diseases and cancers will contribute to the development of novel treatment strategies. In this review, we have presented a review on the development of the understanding on neutrophil heterogeneity from the traditional perspective and a high‐resolution viewpoint. A growing body of evidence has confirmed the double‐edged role of neutrophils in inflammatory diseases and tumors. This may be due to a lack of precise understanding of the role of specific neutrophil subsets in the disease. Thus, elucidating specific neutrophil subsets involved in diseases would benefit the development of precision medicine. Thusly, we have summarized the relevance and actions of neutrophil heterogeneity in inflammatory diseases and cancers comprehensively. Meanwhile, we also discussed the potential intervention strategy for neutrophils. This review is intended to deepen our understanding of neutrophil heterogeneity in inflammatory diseases and cancers, while hold promise for precise treatment of neutrophil‐related diseases.

## Graphical abstract

Schematic representation of the neutrophil heterogeneity in inflammatory diseases and cancer (in blue). This figure is created with BioRender.com.

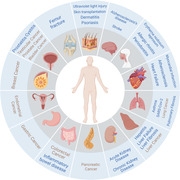

## 1 INTRODUCTION

Neutrophils are important immune cells, defensing against microbial infections and eliminating pathogenic bacteria.^1^ Differentiated and developed in the bone marrow, neutrophils would release and migrate into the blood or tissues, accounting for approximately 50−70% of circulating leukocytes.^2^ Various antimicrobial enzymes are found in neutrophils, including myeloperoxidase, acid hydrolytic enzymes, and elastase.^3^ As a result of being stimulated by the products of pathogenic bacteria, neutrophils would produce antimicrobial enzymes and display their defensive capabilities. Aside from its important defensive role in combating infections, neutrophils could also trigger inflammatory responses at the site of infection, which in turn lead to immunopathological changes.^4^, ^5^ Therefore, it is important to clarify the double‐edged role of neutrophils in the body.

Studies in mounting numbers have identified that phenotypically and functionally distinct neutrophil subtypes that exist in circulating or tissue neutrophils under physiological or pathological conditions.^6^ In recent years, with the development of single‐cell multiomics sequencing technology and mass cytometry, the accurate and unbiased classification of neutrophils has greatly facilitated our understanding of neutrophil heterogeneity.^7^, ^8^ A growing number of researchers are finding that the role of different neutrophil subsets in disease may be complex, as some subsets can contribute to the disease process while others would inhibit disease progression.^9^ The dual role of neutrophil heterogeneity in inflammatory diseases and cancers has gained increasing attention.^10^, ^11^ It is hoped that elucidating the role of neutrophil heterogeneity in diseases would lead to a new era of precision medicine in inflammation and cancer‐related diseases.

**FIGURE 1:**
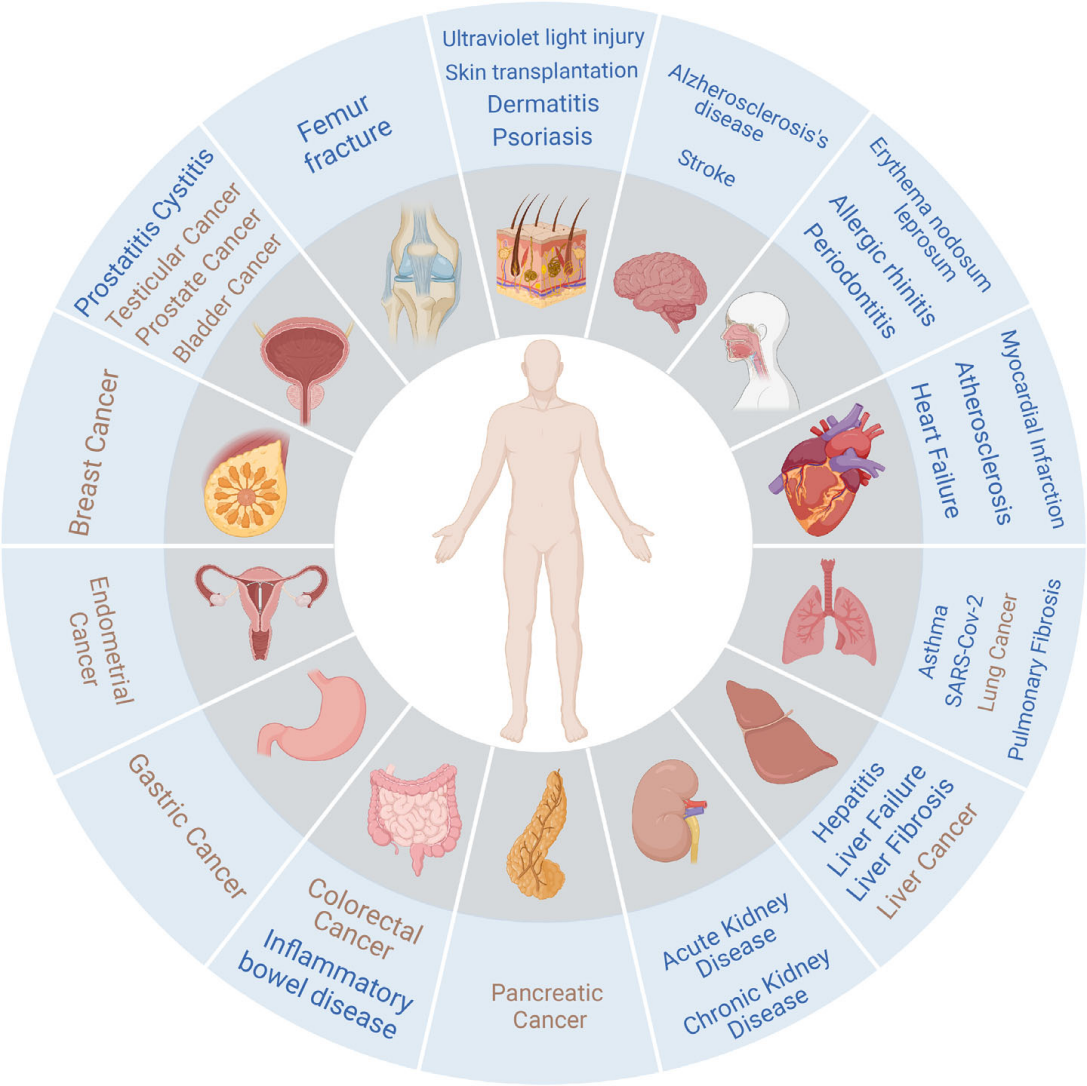
Schematic representation of the neutrophil heterogeneity in inflammatory diseases and cancers (in blue). This figure is created with BioRender.com.

Therefore, this review aims to highlight the double‐edged role of neutrophils in diseases, systematically summarizing the important roles of neutrophil heterogeneity in inflammatory diseases and cancers. Meanwhile, the potential intervention strategy for neutrophils has been discussed. Overall, this review is intended to deepen our understanding of neutrophil heterogeneity in inflammatory diseases and cancers, holding promise for precise treatment of neutrophil‐related diseases (Figure 1).

## 2 THE FUNCTIONS AND THE DOUBLE‐EDGED ROLE OF NEUTROPHILS

### 2.1 Neutrophils in homeostasis

Neutrophils have long been considered as an important part of the innate immune system, playing a pivotal role in controlling infectious diseases.^12^ In physiological context, inactivated neutrophils move slowly in the circulation. The origin of neutrophils is derived from hematopoietic cords located at venous sinuses in the bone marrow and derive from a common committed myeloid progenitor cell.^1^ The neutrophils development are stimulated by transcription factors, proteins, and receptors such as Egr1, HoxB7, S100A8, S100A9, N‐formyl‐methionyl‐leucyl‐phenylalanine receptor and granulocyte‐macrophage colony stimulating factor receptor.^13^, ^14^, ^15^, ^16^ The neutrophil maturation process consists of the following steps: myeloblast→promyelocyte→myelocyte→metamyelocyte→band cell→polymorphonuclear segmented cell. In general, granulocyte colony stimulating factor (G‐CSF) directs the commitment of progenitor cells to the myeloid lineage, promotes proliferation of neutrophil precursors, reduces the transit time through the compartment, and releases of mature neutrophils out of the bone marrow. Once maturation has been completed in the bone marrow and the pool of mature cells is released into the circulation, neutrophils circulate with a set of preformed adhesion and chemotactic receptors and effector proteins to rapidly migrate and respond to multiple microbial and sterile challenges.^17^

### 2.2 Neutrophils in response to stimulation

Upon stimulation by external signals, these neutrophils move rapidly toward the injury site and effect in eliminating pathogenic bacteria and phagocytosis of injured cells.^18^, ^19^ Neutrophils and their derivatives, such as cytokines, reactive oxygen species (ROS), and neutrophils extracellular traps (NETs), are involved in both innate and adaptive immune responses, influencing the function of other immune cells in various ways and thus participate in the disease process.^18^ Neutrophils and NETs could promote macrophage release cytokine while enhancing dendritic cells recruitment and antigen presentation.^20^ Furthermore, by regulating cytokine and growth factor production, neutrophils would promote innate immune resolution and repair. In addition, neutrophils might trigger B cell expansion and antibody production.^21^ Neutrophils and their derivatives could also affect T cell cytokine production and differentiation, thereby influencing T cell function.^22^ During the process of infection or tissue damage, a diverse array of signals could mediate neutrophil activation and forward migration toward the site of damage. Many studies have found that blocked recruitment of neutrophils would lead to the spread of pathogens to the blood and vital organs, resulting in systemic infection and even death.^23^, ^24^, ^25^ Important signals establishing chemoattractant gradients that could take effect in neutrophil recruitment and migration include pathogen‐associated molecular patterns and/or damage‐associated molecular patterns (DAMPs), hydrogen peroxide (H_2_O_2_), fMet‐Leu‐Phe (fMLP), lipid mediators, chemokines, and so on.^4^, ^5^, ^6^ As early signals, DAMPs have been reported to be responsible for early neutrophil recruitment. DAMPs could be sensed by pattern recognition receptors (PRRs), including Toll‐like receptors (TLRs) and NOD‐like receptors.^26^, ^27^ They would bind to and activate G‐protein‐coupled receptors (GPCRs) on neutrophils, thereby inducing resident cells to produce inflammatory mediators and to modulate neutrophil migration, function and behavior.^28^ N‐formyl peptides, such as fMLP, is another early signal which could trigger neutrophil chemotaxis and activation.^29^, ^30^, ^31^ Derived from bacterial proteins or released from mitochondria after tissue damage, they would activate human neutrophils by binding to the GPCRs FPR1, FPR2, and FPR3. H_2_O_2_ could promote chemotaxis of human and mouse neutrophils.^32^, ^33^ At present, SRC family kinases (SFKs) have been reported to be a direct sensor to H_2_O_2_, required for neutrophils.^32^ Interestingly, the peak of H_2_O_2_ production occurred around 30 min after injury, and the signal was completely abolished by myeloperoxidase activity of injury‐associated neutrophils 1 h later.^34^ Chemokines and lipid mediators usually cause long‐term chemotactic signaling in order to facilitate more sustained recruitment and migration of neutrophils.^35^, ^36^, ^37^ Many studies have reported the involvement of chemokines in neutrophil migration, including CXCL1, CXCL2, CXCL3, CXCL5, CXCL6, CXCL7, and CXCL8.^38^, ^39^, ^40^ These chemokines may be derived from immune and nonimmune cells during tissue infection and injury.^41^, ^42^ Neutrophils would sense these chemokines through the GPCRs, CXCR1, and CXCR2,^43^, ^44^, ^45^ leading to downstream signaling activation of vasodilator‐stimulated phosphoprotein, phosphoinositide 3‐kinase, and SFKs, contributing to neutrophil‐directed migration.^46^, ^47^ Lipid mediators, including leukotriene B4 (LTB4), 5‐oxo‐ETE, and 5‐KETE, derived from arachidonic acid, could induce neutrophil chemotaxis.^48^, ^49^, ^50^ During the process of chemotaxis, neutrophils must integrate multiple chemical signals, respond to physical constraints, and prioritize their directional decisions to generate an efficient immune response. A recent study reported that neutrophils were more likely to choose paths with the steepest chemoattractant gradient and the most direct approach angle, and the migration efficiency across planar chambers was inversely correlated with chamber diameter.^51^ Many studies have focused on the effect of specific molecules on the migration efficiency of neutrophils. For example, G‐protein‐coupled receptor GPR35 was upregulated in activated neutrophils, and it promoted their migration.^52^ Another study reported that CXCL1, derived from endothelial cells, and CXCL2, which was derived from neutrophils, acted in a sequential manner to guide neutrophils through venular walls.^53^ In addition, many other molecules, including filamin A, ICAM‐1, IL‐8, G protein signaling 5 (RGS5), Glia maturation factor‐gamma, and MMP2, have also been reported to be involved in affecting the efficiency of neutrophil migrate.^54^, ^55^, ^56^, ^57^, ^58^ At the site of damaged or infected tissue, neutrophils would fight against pathogens mainly through phagocytosis and production of ROS, bactericidal granulocytes, and NETs.^59^ We would focus on the specific effects of neutrophils in the context of different diseases. After the acute inflammatory phase, neutrophil resolution has been shown to be critical in preventing tissue damage and transition to chronic wounds.^60^ Neutrophil resolution would occur through neutrophil apoptosis or necrosis, with subsequent clearance by macrophages.^61^ Interestingly, some studies have reported that neutrophils would leave the site of damaged or infected tissue in a process termed neutrophil reverse migration.^5^, ^61^ Wang et al.^62^ reported that neutrophils can migrate from the site of inflammation to the blood vessels, and then subsequently return to the bone marrow to promote apoptosis. This process has been known as reverse migration and could also occur through reverse transendothelial migration.^63^ Neutrophils could leave the wound in other ways as well, such as metastasis from adjacent tissues and lymphatic metastasis, and eventually spread to other parts of the body.^64^ The reverse migration of neutrophils played a crucial role in accelerating the resolution of inflammation.^65^ However, another study reported that the return of neutrophils from the wound site to the blood vessel might contribute to the spread of systemic inflammation.^66^ This contradiction may be caused by different disease backgrounds. The possible mechanisms of neutrophil reverse migration involved adhesion molecule C (JAM‐C)‐mediated abnormal endothelial cell function, HIF signaling pathway mechanism, lipid mediating mechanism, and CXCL12/CXCR4 signal axis mechanism.^67^, ^68^, ^69^, ^70^ In addition, macrophages could also promote neutrophil reverse migration.^71^ Moreover, a recent study highlighted the importance of vascular permeability in neutrophil reverse migration.^72^ Several studies have described the important role of neutrophil swarming in diseases.^73^, ^74^ Neutrophil swarming is the formation of a population of neutrophils in infected tissues. The coordination of this population response is essential for the removal of bacteria.^75^ As reported, a cell‐intrinsic stop mechanism for the self‐organization of collective behavior suggested the crucial role of GPCR desensitization in attenuating the self‐organized swarming dynamics of neutrophils. In detail, when neutrophils sense high concentrations of swarm‐secreted attractants (LTB4 and CXCL2), the GPCR kinase GRK2 desensitizes the corresponding GPCRs to induce migration arrest.^73^ This is a critical step for tissue repair after infection. To date, however, the exact mechanism of neutrophil reverse migration remains unclear. Further research is required to determine the effect of neutrophil reverse migration on different diseases and how to accurately intervene.

### 2.3 The double‐edged role of neutrophils

Neutrophils play a complex role in diseases, with the ability to both promote and inhibit disease progression. They can promote tumor growth and metastatic progression through the secretion of tumor‐promoting cytokines, enhancement of tumor angiogenesis, modulation of the extracellular matrix, support of tumor cell dissemination, priming of the premetastatic niche, and suppression of antitumor immune responses.^76^, ^77^, ^78^ However, neutrophils can also limit tumor growth and metastatic progression through direct cytotoxicity, antibody‐dependent cell‐mediated cytotoxicity, and inducing adaptive antitumor immunity.^79^, ^80^, ^81^, ^82^ The double‐edged role of neutrophils in diseases, both promoting and suppressing diseases, has garnered attention from researchers, with most recent studies highlighting the existence of different subsets of neutrophils with varying functions in blood or tissues.^83^, ^84^ Neutrophils exhibit heterogeneity in both physiological and pathological conditions.^83^ Specific signals at different developmental stages or disease backgrounds would drive neutrophil heterogeneity.^9^ We will discuss this in detail in the context of different diseases. Clarification of neutrophil heterogeneity may help explain the dual role of neutrophils in diseases, and understanding the complicated role of neutrophils might benefit the development of treatment for a great many diseases. Therefore, in this review, we focus on how to analyze neutrophil heterogeneity and its double‐sided role in the course of inflammatory diseases and tumors.

## 3 METHODS OF STUDYING NEUTROPHIL HETEROGENEITY

**FIGURE 2:**
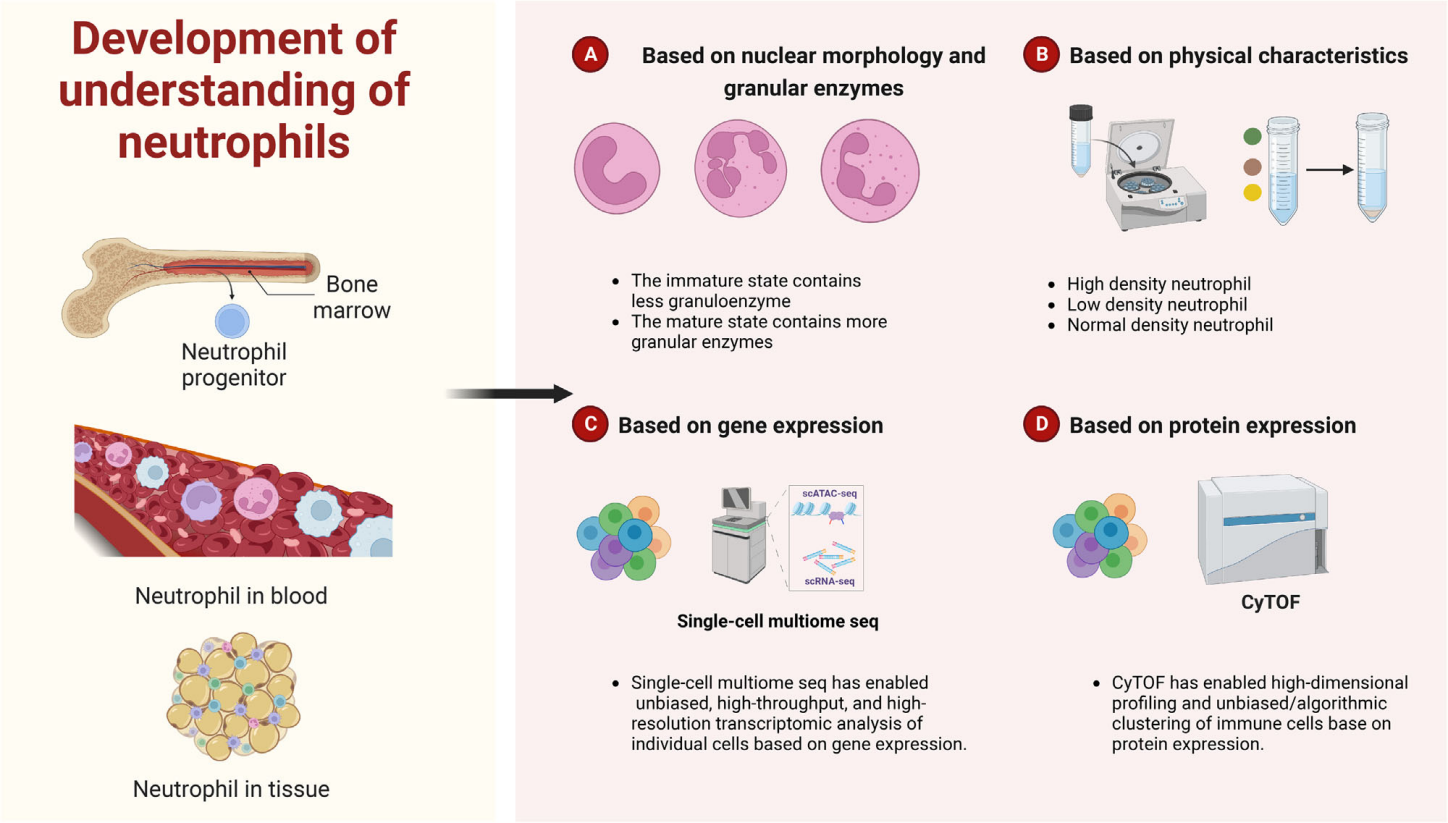
Development of understanding of neutrophils. This figure is created with BioRender.com.

The advent of high‐resolution methods coupled with investigations of traditional methods in recent years has helped us to reveal the presence of heterogeneity in neutrophils, as presented in various phenotypes, under physiological and pathological contexts^83^, ^85^, ^86^ (Figure 2). Neutrophils from different subpopulations function diversely in inflammatory diseases and cancers, which merits further exploration.^87^, ^88^, ^89^

### 3.1 Studies on neutrophils by the traditional methods

Neutrophil heterogeneity has historically been defined as high‐ and low‐density neutrophils (HDNs and LDNs) through its physical properties.^90^, ^91^, ^92^, ^93^ This easy classification of cells has resulted in the retention of this widely accepted definition, despite the lack of information at high resolution.^90^, ^93^ It is generally accepted that LDNs are immature neutrophils containing few granule enzymes, whereas HDNs are mature neutrophils with higher levels of granule enzymes.^10^, ^83^, ^94^, ^95^, ^96^ However, complex transitions between these two subpopulations occur in pathological scenarios.^97^, ^98^ Neutrophils often undergo changes in their nucleus and surface marker genes as they mature and develop.^10^, ^99^ There are three neutrophil subsets in the mouse bone marrow, including a committed proliferative precursor terming preneutrophils, which would sequentially differentiate into nonproliferating immature and mature neutrophils. Preneutrophils express CD177, whereas differential expression of C‐X‐C chemokine receptor 4 (CXCR4), CXCR2 and CD101 allows for discrimination between immature neutrophils (which are CXCR2^−^CD101^−^) and mature neutrophils (which are CXCR2^+^CD101^+^).^99^, ^100^, ^101^ As neutrophils mature, surface expression of markers gradually increases, such as CD62L, CD101, or glycosylphosphatidylinositol‐linked receptor Ly6G in mice.^83^, ^94^ Physiologically, neutrophils would release into the circulation and enter into tissues within a few days, often relying on specific adhesion molecules and regulated by circadian rhythm.^98^, ^102^, ^103^, ^104^, ^105^, ^106^ While under pathological conditions, neutrophils behave in a more complex manner. To be specific, neutrophils die in inflamed or damaged tissues and are even recruited back to the bone marrow by regulating the expression of related receptors.^62^, ^107^ Attempts have also been made to differentiate the functional differences of neutrophils in cancers according to their density.^96^, ^97^, ^98^, ^108^, ^109^ Even so, this relies on non‐high‐resolution information, and it often appears to be inaccurate. As an example, some studies have demonstrated that immature LDNs have the ability to promote cancer progression or immunosuppression.^110^, ^111^, ^112^ While some other studies have suggested that mature neutrophils may also possess these same properties.^97^, ^98^, ^113^ Consequently, researches on neutrophil heterogeneity under non‐high‐resolution conditions have been of limited precision and have substantially limited our understanding.

### 3.2 Studies on neutrophils by high‐resolution methods

Recently, the development of novel technologies, including single‐cell transcriptome sequencing, single‐cell assay for transposase‐accessible chromatin sequencing and mass cytometry, has greatly promoted the accurate and unbiased classification of neutrophils, thus contributing to the advancement of neutrophil heterogeneity.^114^, ^115^, ^116^, ^117^ In humans, mice, non‐human primates, and even other mammals, researchers have examined the heterogeneity of neutrophils derived from bone marrow in depth, laying the foundation for understanding how neutrophils develop.^101^, ^118^, ^119^, ^120^, ^121^, ^122^ Studies have revealed that in healthy individuals, 45−65% of circulating neutrophils are CD177^+^, and about 20−25% of circulating neutrophils express the glycoprotein olfactomedin 4 (OLFM4).^10^, ^123^ The presence of other circulating neutrophil subpopulations, for example, those expressing the T cell receptor αβ (TCRαβ)^+^ or proangiogenic CD49d^+^CXCR4^+^ vascular endothelial growth factor (VEGFR1)^+^, is also observed in healthy individuals.^124^, ^125^ Further evidence has been provided that these circulating neutrophils presented in mice.^126^ In addition, the proportion of CXCR4^−^CD62L^+^ and CXCR4^+^CD62L^−^ circulating neutrophils in healthy individuals followed the diurnal regime. The immature CD66b^+^CD10^−^ neutrophils would generate antibacterial granules, ready to enter into the circulation in response to external signals.^127^ These neutrophils, despite their "immature state," are capable of performing innate immune functions and display functional plasticity, as well as immunoregulatory properties.^128^, ^129^ Physiologically, heterogeneity of circulating neutrophils and neutrophils in tissue is more complex. A more comprehensive discussion would be conducted in the following context relating to multiple diseases. Researchers have developed computational methods to infer cell differentiation trajectories, however, experimental validation has yet to be achieved.^130^, ^131^ In addition, a further study of the heterogeneity of neutrophils across species is also worthwhile.

## 4 NEUTROPHIL HETEROGENEITY IN DISEASES

### 4.1 Neutrophil heterogeneity in inflammation‐related diseases

**FIGURE 3:**
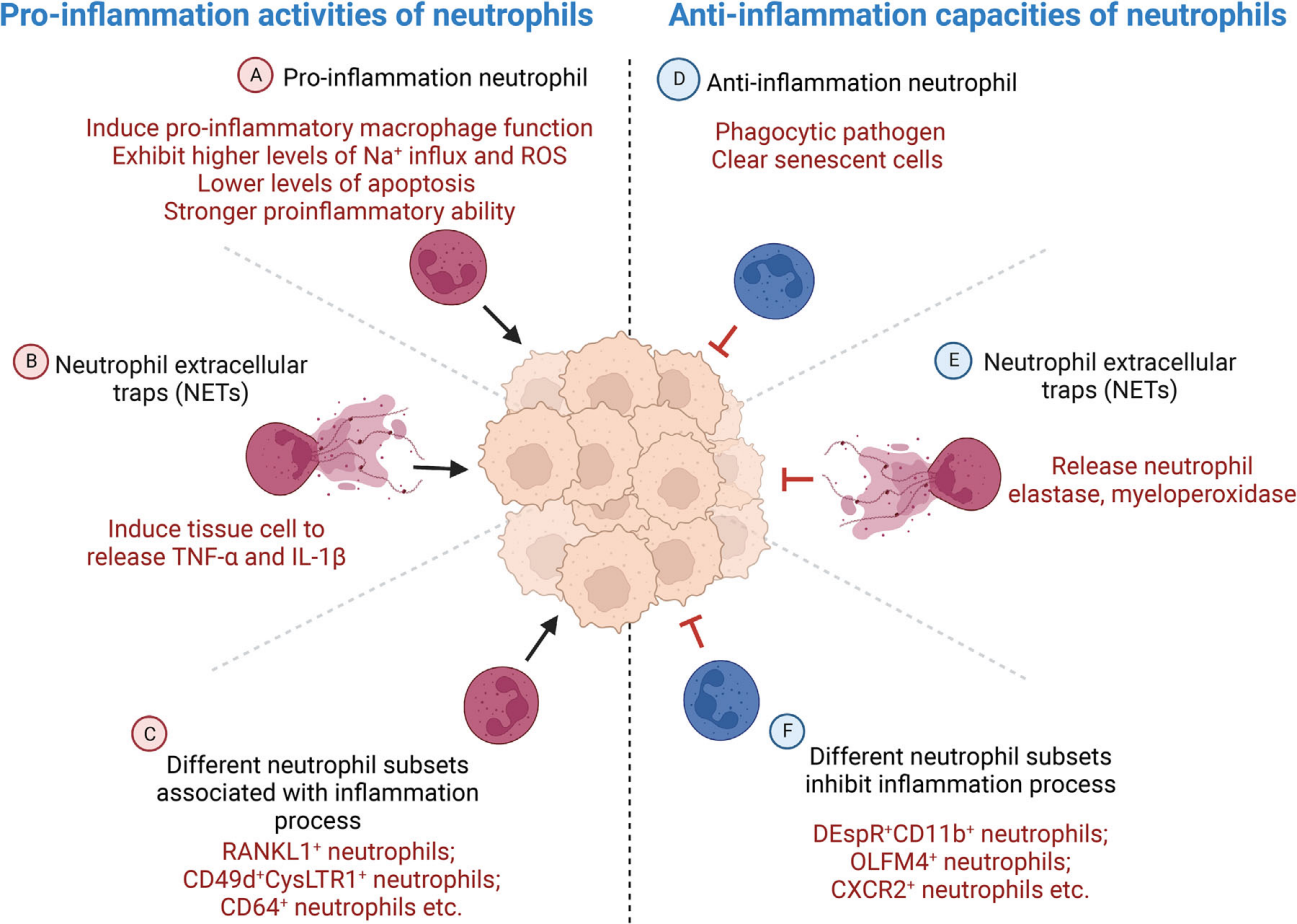
Neutrophils show both proinflammation activity or anti‐inflammation capacities. This figure is created with BioRender.com.

In both acute and chronic inflammation, the genetic structures of neutrophils appear to undergo a reprogramming process due to the stimulation of external signals, resulting in dynamic transitions between subpopulations.^132^, ^133^, ^134^ In inflammatory diseases, infection or ischemia can induce the production of G‐CSF, GM‐CSF, or other myelopoietic factors, which contribute to different functional neutrophils.^135^, ^136^, ^137^ We will discuss what factors would contribute to neutrophil heterogeneity in the context of diseases. There has been evidence that neutrophil plasticity and heterogeneity are involved in the process of various inflammatory diseases, both physiologically and pathologically.^60^ In addition, neutrophils would not only present proinflammation activity via proinflammation neutrophils, neutrophil extracellular traps (NETs), and different neutrophil subsets linked with inflammation process, but also hold anti‐inflammation capacities through various pathways, including anti‐inflammation neutrophils, NETs, and neutrophil subsets that would inhibit inflammation process (Figure 3). Therefore, analyzing neutrophil heterogeneity is of great significance in the field of inflammation‐related diseases, including liver diseases, kidney diseases, respiratory diseases, cardiovascular diseases, and inflammatory bowel diseases (IBDs).

#### 4.1.1 Liver diseases

Liver diseases, including alcoholic liver injury, nonalcoholic fatty liver disease, liver fibrosis, and liver failure, pose serious threats to human life. Neutrophils function vitally in the above‐mentioned diseases.^138^, ^139^ The accumulation of neutrophils in liver tissue promotes liver injury has been reported by multiple research teams.^140^, ^141^, ^142^ Senger and coworkers^143^ found that blocking dipeptidase‐1 on endothelial cells could reduce the recruitment of neutrophils to liver, improving inflammation in liver tissues. Thus, reducing the accumulation of neutrophils in liver tissues could alleviate liver injury, offering new hope for clinical treatment of liver injury. Hwang et al.^144^ also reported that the overexpression of C‐X‐C motif chemokine ligand 1 (CXCL1) in mice liver drive steatosis‐to‐nonalcoholic steatohepatitis (NASH) progression through neutrophil‐derived ROS and activation of stress kinases, and this process could be reversed by IL‐22 treatment. Interestingly, another study showed that the absence of neutrophils contributes to resolution of liver inflammation, a process associated with neutrophil‐induced proinflammatory macrophage function.^145^ It is likely that neutrophils play different roles for they function distinctively in varied disease stages. HDNs and LDNs exist in liver tissues from clinical samples and alcoholic liver injury mice. Alcohol could induce HDNs to form NETs, thereby leading LDNs to reside in the liver and escape been cleaned‐up by macrophages, causing chronic inflammation.^146^ Kolodziejczyk et al.^147^ reported the presence of two neutrophil subpopulations in the liver tissue of a mouse model of acute liver failure, among which CXCL2^+^ neutrophils may be involved in disease progression through their proinflammatory function. In addition, the progression of fibrosis in patients with chronic viral hepatitis has been shown to be associated with IL‐17(+) neutrophils.^148^ At present, the liaison between some neutrophil subpopulations and liver‐related diseases has been analyzed. though, the causal relationship between these specific neutrophil subpopulations and diseases remains unclear, warranting further investigation.

#### 4.1.2 Kidney diseases

Glomerulonephritis is a common concomitant of systemic lupus erythematosus (SLE).^149^, ^150^ The proportion of neutrophils peripheral blood neutrophils and in kidneys is significantly increased in SLE patients and mice.^151^ Among them, LDNs exhibited higher activity.^152^ Inhibition of infiltrating neutrophils in the kidney ameliorated lupus nephritis progression in mice.^152^ Neutrophils also play an important role in other acute and chronic nephritis progression.^153^, ^154^ Neutrophil subpopulations differ functionally in various kidney diseases. To be specific, the presence of OLFM4^+^ neutrophils have a negative impact on sepsis‐induced kidney injury, which is associated with renal cell apoptosis and increased plasma creatinine levels.^155^ Skopelja‐Gardner et al.^156^ reported that acute skin exposure to ultraviolet light drives kidney injury via Ly6G^+^GFP^+^ICAM1^hi^CXCR1^lo^ neutrophils. Another study showed an increase in the proportion of CD14^−^CD16^−^CD15^+^ neutrophils in the peripheral blood of patients with chronic nephritis, and the function of this subset of neutrophils is mainly related to vascular calcification.^157^ These findings have further confirmed the significance of neutrophil heterogeneity in nephritis.^89^, ^158^ Targeting neutrophil elastase has been proven to alleviate chronic nephritis.^159^ Nevertheless, the deficiency of efficient drugs that target particular neutrophil subsets exists, requiring additional exploration. There are relatively few studies on neutrophil heterogeneity in renal tissues, and the lack of functional experimental studies on specific neutrophil subsets also warrant further investigation.

#### 4.1.3 Respiratory diseases

The essential role of neutrophils in diseases, including pneumonia, asthma, and chronic obstructive pulmonary disease (COPD), has gradually aroused researchers’ attention.^160^, ^161^, ^162^ During acute lung infection, neutrophils are the first to be recruited in large numbers to the infection site among all cell types, thereby participating in the initial clearance of infected cells. However, a recent study reported that impairing alveoli outnumber alveolar macrophages chemotaxis toward bacteria would induce superfluous neutrophil recruitment, leading to inappropriate inflammation and injury.^163^ This illustrates the double‐edged role of neutrophils in respiratory disease. An increasing number of studies in recent years have established the importance of neutrophil heterogeneity in respiratory diseases.^164^, ^165^ Specifically, the phenotype of neutrophils alters as a disease progresses.^166^ The number of CD177^+^ neutrophils, CD64^+^ neutrophils and OLFM4^+^ neutrophils is significantly increased in the peripheral blood of patients with sepsis and asthma, which has been confirmed by several studies.^167^, ^168^, ^169^, ^170^, ^171^ The CD16^high^ CD62L^dim^ neutrophils were unveiled to enhance the response to bradykinin in both human isolated small airways and murine tracheae.^172^ In hemorrhagic shock and sepsis mouse, inhibition of OLFM4 attenuated lung inflammation in mice.^155^, ^173^ Acute and chronic respiratory inflammation would mediate the emergence of neutrophil subsets.^174^, ^175^ Another study revealed the impaired activation and phagocytosis functions of CD123^+^ neutrophils and PD‐L1^+^ neutrophils in the peripheral blood of sepsis patients. Subsets that highly express PD‐L1 exerted an immunosuppressive effect, including the inhibition of T cell activation, induction of T cell apoptosis and trans‐differentiation, while the number of CD123^+^ neutrophils correlated with the severity of the disease.^176^, ^177^ In a sepsis mouse model, extracellular cold‐inducible RNA‐binding protein (eCIRP) can induce the formation of the proinflammatory phenotype Ly6G^+^ CD11b^hi^ of LDNs through TLR4.^178^ In a mice model of chronic granulomatous pneumonia, G‐CSF mobilized immature CD101^neg^ neutrophil subsets released from the marrow with a proinflammatory phenotype.^179^ From the single‐cell scale, researchers have systematically described the heterogeneous population and transcriptome dynamics of neutrophils in the process of maturation, differentiation, and fate determination in both homeostatic and inflammatory states. In the inflammatory state, the heterogenous population of neutrophils did not change, while the proportion, functional characteristics, expression of the transcription factor, and the transformation pathway altered dramatically between populations. This provides basic information for the precise investigation of neutrophil heterogeneity.^180^, ^181^ It was reported that, compared with normal lung tissue, two distinct neutrophil subsets were generated in *Cryptococcus neoformans*‐infected lung tissue, and these two subsets highly expressed inflammation‐related genes and oxidative stress‐related genes, respectively. Neutrophils highly expressing inflammation‐related genes can modulate cell‐cell communication with dendritic cells and alveolar macrophages.^182^ CD49d^+^CysLTR1^+^ neutrophils were present in the nasal lavage fluid from patients with viral respiratory tract infections.^183^ A significantly increased amount of CD63^+^ neutrophils was detected in the blood of cystic fibrosis patients.^184^ Both PD‐L1^hi^ and PD‐L1^lo^ neutrophils were present in sputum from cystic fibrosis patients, while in healthy control, only PD‐L1^hi^ neutrophils were present.^185^ Moreover, RANKL1^+^ neutrophils were found specifically present in the peripheral blood of COPD patients.^186^ The pivotal role of neutrophil heterogeneity in Coronavirus disease 2019 (COVID‐19) has lately received increasing attention.^187^, ^188^, ^189^ In a recent study, interferon (IFN)‐stimulated gene (ISG) neutrophils were shown absent in the lung tissue of patients with severe COVID‐19.^190^, ^191^ Immature and PD‐L1^+^ neutrophils were, in contrast, significantly increased.^192^ Other subpopulations, such as CXCR4^high^/CD62L^low^ neutrophils and CD16^dim^/CD62L^bright^ neutrophils, have also been reported to be involved in the pathogenesis of COVID‐19, which can suppress T cell function.^113^, ^193^ The beneficial effects of dexamethasone during COVID‐19 were mainly through the downregulation of IFN‐responsive genes and activation of IL‐1R2^+^ neutrophils.^194^ Unlike COVID‐19 infection, the proportion of ICAM‐1^+^ neutrophils in the peripheral blood of SARS‐CoV‐2‐infected patients varied vastly.^195^ This phenotype may be induced by CIRP.^196^ Carstensen et al.^197^ demonstrated that inhibition of the DEspR^+^CD11b^+^ neutrophil subset could alleviate multiple respiratory diseases.^198^ While targeting the intervention of CD11b^+^CD18^+^ neutrophils would aggravate influenza‐induced lung damage, the specific mechanism of which was related to affecting functions of T cells.^199^ Some researchers have speculated that neutrophil differentiation in humans and mice are similar before and after lung inflammation through computational analysis.^200^ This, to some extent, indicates that conclusions from mouse‐based studies could provide support for clinical research. The progression of inflammatory diseases usually results from a combination of interactions between neutrophils and other immune cells. At present, the interactions between specific subsets of neutrophils and other immune cells are relatively rare, which is worthy of further investigation.

#### 4.1.4 Cardiovascular diseases

The study of neutrophils, especially neutrophil heterogeneity, is of great momentousness in the field of cardiovascular and cerebrovascular diseases.^201^, ^202^, ^203^ The normal‐density neutrophils (NDNs) and LDN subpopulations in the peripheral blood of patients with hypertension‐associated chronic inflammation exhibited higher levels of Na^+^ influx and ROS, along with lower levels of apoptosis and stronger proinflammatory ability, thereby promoting the chronic inflammatory response associated with hypertension.^204^ Some research teams identified that Ly6G^+^SiglecF^+^ (Myc^+^NFϰB^+^) neutrophils served as proinflammatory cells in the progression of myocardial infarction (MI).^205^, ^206^ The CD33^high^CD16^low^ neutrophils were the main effector neutrophil subset in the peripheral blood of MI patients.^207^ Interestingly, compared with young male mice, reduced neutrophil proinflammatory gene expression were found in the infarct region of young females.^208^ This suggested that sex differences existed in the function of neutrophils. The proportion of IFN‐responsive/ISG‐expressing neutrophils in the peripheral blood of MI patients was increased. With the help of experiments on mouse models, the researchers indicated that this group of neutrophils originated from the bone marrow and was negatively regulated by Nrf2 activation.^209^ In the mouse model of heart failure, two neutrophil subpopulations with different expression patterns were found within the myocardial. Despite the significant increase in the proportion and both having proinflammatory function, the two populations have functional differences in antigen presentation.^210^ Patients with ischemic stroke had higher percentages of the overactive senescent (CXCR4^bright^/CD62L^dim^) neutrophil subset and neutrophils with a reverse transendothelial migration (CD54^high^CXCR1^low^) phenotype in their peripheral blood than controls, and this was significantly correlated with disease progression.^211^ Similarly, the ratio of CXCR4^high^/CD62L^low^ neutrophils in the peripheral blood of Parkinson's patients could also reflect the disease process.^212^ Neutrophil‐specific deletion of Syk would improve cognitive dysfunction after traumatic injury to the brain.^213^ Trivedi et al.^213^ reported the existence of a proinflammatory neutrophil subset (antigen‐presenting cell‐like neutrophils) in both hyperlipidemic patients and atherosclerotic mice. This unique phenotype of neutrophils had the ability to activate the adaptive immune response, thereby promoting atherosclerosis progression. Moreover, a positive correlation between the presence of neutrophils and CD3^+^ T cells was observed.^214^ Despite the fact that many studies have confirmed the diversity of neutrophils in peripheral blood in cardiovascular and cerebrovascular diseases, there is insufficient evidence for the heterogeneity of neutrophils in situ in tissues, which may be attributed to the difficulty in obtaining clinical samples.

#### 4.1.5 Inflammatory bowel diseases

IBDs are chronic inflammatory conditions affecting the digestive system.^215^ Abnormal function of neutrophils significantly affects the progression of IBDs.^216^ Neutrophils and NETs play a pivotal role in IBD.^217^ There was upregulation of NETs in the tissues of patients with ulcerative colitis (UC), and NETs could activate the UC lamina propria mononuclear cells to activate extracellular signal‐regulated kinase‐1/2 (ERK1/2), thereby enhancing TNF‐α and interleukin‐1β (IL‐1β) production.^218^ Neutrophils exhibit both cytotoxicity and the potential to cause severe tissue damage to the colon tissue. They are also capable of defensive functions. NETs in UC tissues, for instance, have been shown to be advantageous in terms of immune thrombus formation and the prevention of colonic bleeding.^219^ The remarkable effect of improving IBD by affecting neutrophil function has gradually attracted researchers’ interest.^220^ The artemisinin analogue SM934 or the Annickia polycarpa extract ameliorated UC in mice by inhibiting neutrophil activation or recruitment.^221^, ^222^ CD177^+^ neutrophils played a role in protecting intestinal barrier function in IBD by increasing bactericidal activity and IL‐22 production.^223^, ^224^ Therefore, targeting CD177^+^ neutrophils may benefit the treatment of IBD. IL‐17^+^ neutrophils in the colons of mice with dextran sulfate sodium‐induced colitis might contribute to the progression of the disease.^225^ Levinsky et al.^226^ demonstrated that OLFM4^+^ neutrophils centrally participated in the pathologic pathway leading to intestinal ischemia/reperfusion injury in mice through experiments involving adoptive transfer of bone marrow. In the realm of IBD drug therapy, the existence of certain neutrophil subsets was also essential. Due to the presence of CXCR2^+^ neutrophils, patients with UC often appeared insensitive to ustekinumab.^227^ Thus, interventions targeting specific neutrophil subsets in colon tissue are expected to pave the way for precise treatment for IBD.

#### 4.1.6 Other inflammatory diseases

**FIGURE 4:**
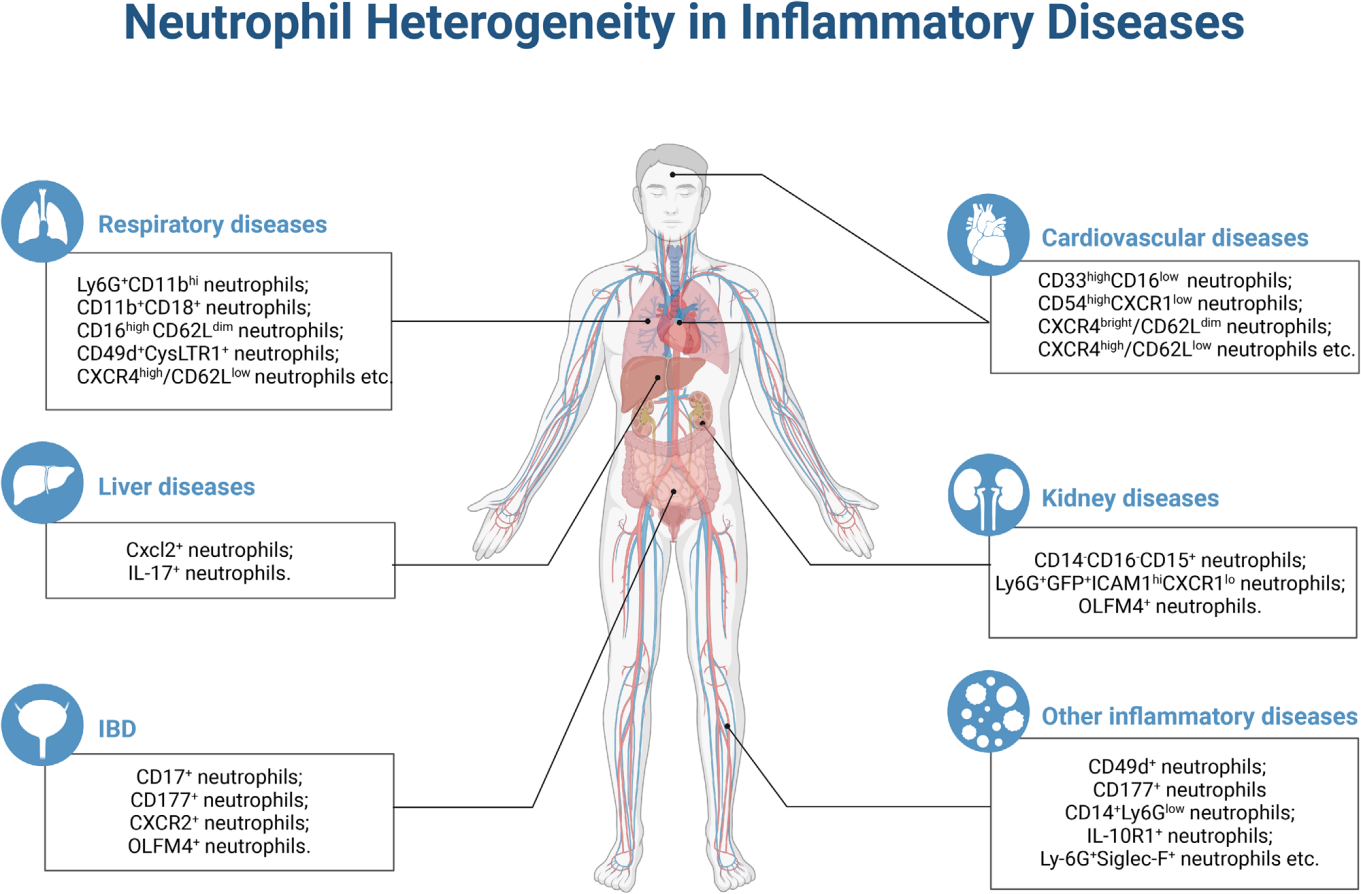
Neutrophil heterogeneity in inflammatory diseases. This figure is created with BioRender.com.

In addition to the above‐mentioned prevalent acute and chronic inflammatory diseases mentioned above, neutrophil heterogeneity is also observed in other inflammatory diseases. Hypodense neutrophils and the ability to form NETs in peripheral blood and gastrointestinal tissues in patients with idiopathic inflammatory myopathy and vasculitis were greatly upregulated.^228^, ^229^ Neutrophil subpopulations also have fundamental effects on periodontal health regulation, as CD177^+^ neutrophils were recruited to gingival crevicular fluid in periodontitis preferentially.^230^, ^231^, ^232^ Some researchers have identified specific neutrophil subsets, such as CD14^+^Ly6G^low^ neutrophils, that could secrete growth factors NGF and IGF‐1, thereby promoting neuronal survival.^233^, ^234^ Ly‐6G^+^SiglecF^+^ neutrophils specifically exist in the allergic mouse nasal mucosa, exhibiting an activated phenotype.^235^ IL‐10R1^+^ neutrophils were found in the peripheral blood of patients with erythema nodosum leprosum. Senescent CD10^neg^CD16^low^CD11b^low^ and immature CD10^neg^CD16^neg^CD11b^neg^ neutrophils in psoriatic skin tissue induced more IL‐17 expression.^236^ Induction of CD49d^+^ neutrophil production favors wound closure after skin transplantation.^237^ CD11b^high^/CD11a^high^ neutrophils specifically appeared in peripheral blood in a mouse fracture model.^238^ In addition, common IFN‐responsive neutrophils played crucial roles in viral infection and other diseases. In summary, numerous studies have elucidated the essential function of neutrophil heterogeneity in both acute and chronic inflammatory diseases, including liver diseases, kidney diseases, respiratory diseases, cardiovascular and cerebrovascular diseases, and colon diseases^4^, ^18^, ^239^ (Figure 4). However, there is currently limited research on the detailed functions of neutrophil subsets in disease development, especially the lack of functional verification of neutrophil subsets in vivo. This represents a potential avenue for future research (Table 1).

**TABLE 1:** Neutrophil heterogeneity in inflammation‐related diseases.

Diseases	Species	Samples	Neutrophil heterogeneity	Functions	References
Alcohol‐associated hepatitis	Human/mice	Liver tissues	High‐density neutrophils (HDNs); low‐density neutrophils (LDNs)	HDNs contribute to NETs; LDNs lead to persistent inflammation	^146^
Acute liver failure	Mice	Liver tissues	CXCL2^+^ neutrophils	Regulatory cell infiltration	^147^
Chronic viral hepatitis	Human	Liver tissues	IL‐17^+^ neutrophils	The number of cells increases significantly in the fibrotic stage	^148^
Sepsis‐related kidney injury	Human/mice	Blood, kidney tissues	OLFM4^+^ neutrophils	Juvenile OLFM4‐null mice are protected from sepsis‐related injury	^155^, ^173^
Kidney inflammation	Mice	Kidney	Ly6G^+^GFP^+^ICAM1^hi^CXCR1^lo^ neutrophils	Promote inflammation	^156^
Chronic kidney disease	Human	Blood	CD14^−^CD16^−^CD15^+^ neutrophils	Regulatory vascular calcification	^157^
Septic shock	Human	Blood	OLFM4^+^ neutrophils	The number of cells increases significantly in patients	^167^, ^171^
Septic shock	Human	Blood	CD177^+^ neutrophils	The number of cells increases significantly in patients	^168^, ^169^
Airway hyperreactivity	Human/mice	Blood	CD16^high^ CD62L^dim^ neutrophils	To enhance the response to bradykinin in both human isolated small airways and murine tracheae	^172^
Sepsis	Mice	Bone marrow	PD‐L1^+^ neutrophils	Play an immunosuppressive role in infection	^176^
Sepsis	Human	Blood	PD‐L1^+^ neutrophils, CD123^+^ neutrophils	The proportion of CD123^+^ neutrophils correlated with clinical severity	^177^
Sepsis	Mice	Blood	Ly6G^+^CD11b^hi^ neutrophils	Promote inflammation	^178^
Chronic granulomatous disease	Mice	Bone marrow	CD101^neg^ neutrophils	Trafficked to the lung and acquired a significantly more proinflammatory transcriptome	^179^
Bacterial infection	Mice; human	Spleen; blood	Interferon‐stimulated gene (ISG) neutrophils; Ly6G^low^ CXCR4^hi^ cells; Ly6G^hi^ CXCR4^hi^ cells	The number of ISG neutrophils increases significantly in spleen of human and mice during infection; the number of Ly6G^low^ CXCR4^hi^ cells and Ly6G^hi^ CXCR4^hi^ cells increases significantly in blood and spleen during infection	^180^
SARS‐CoV‐2 infection	Non‐human primates	Lung tissue, bronchoalveolar lavage fluid	CD11b^+^neutrophils	Reflect the severity of the disease	^181^
Cryptococcus neoformans (Cn) infection	Mice	Lung	Ox‐PMN; Cyt‐PMN	Ox‐PMNs interact with the fungus and generate ROS; Cyt‐PMNs respond to cytokines to modulate cell‐cell communication with dendritic cells and alveolar macrophages	^182^
Acute onset of upper respiratory symptoms	Human	Nasal lavage	CD49d^+^CysLTR1^+^ neutrophils	The number of cells increases significantly in patients	^183^
Cystic fibrosis	Human	Blood	CD63^+^ neutrophils	The number of cells increases significantly in patients	^184^
Cystic fibrosis	Human	Sputum	PD‐L1^high^ neutrophils	T cell suppression	^185^
COPD	Human	Blood	RANKL1^+^ neutrophils	Correlate with IL‐1β, IL‐6 and IL‐8 in plasma in COPD	^186^
SARS‐CoV‐2 infection	Human	Bronchoalveolar lavage fluid	CD16^Int^ neutrophils	Promote inflammation	^187^
SARS‐CoV‐2 infection	Human	Blood	DEspR^+^ neutrophils	Correlate with elevated circulating CCL23, increased NETosis, and the severity of the disease	^189^, ^198^
SARS‐CoV‐2 infection	Human	Blood	ISG neutrophils	The number of cells decreases significantly in patients with severe disease	^190^
SARS‐CoV‐2 infection	Human	Blood	CD16^Int^ low‐density neutrophils	Promote T cell proliferation	^191^
SARS‐CoV‐2 infection	Human	Blood	PD‐L1^+^ neutrophils	Immunosuppressive function	^192^
SARS‐CoV‐2 infection	Human	Blood	CXCR4^high^/CD62L^low^ neutrophils	Promote inflammation	^193^
SARS‐CoV‐2 infection	Human	Blood	IL2^+^ neutrophils	Anti‐inflammation	^194^
SARS‐CoV‐2 infection	Human	Blood	ICAM‐1^+^ neutrophils	The number of cells increases significantly in patients	^195^
Acute lung injury	Human, rhesus macaque, rat	Bronchoalveolar lavage fluid	DEspR^+^CD11b^+^/CD66b^+^ neutrophils	The number of cells increases significantly in disease	^197^
Severe influenza virus infection	Mice	Lung	CD11b^+^CD18^+^ neutrophils	Regulate T cell function	^199^
Hypertension	Human	Blood	Normal‐density neutrophils (NDN), low‐density neutrophils (LDN)	Promote inflammation	^204^
Myocardial infarction	Mice	Blood, bone marrow, heart	Ly6G^+^SiglecF^+^ neutrophils; ISG neutrophils	Promote inflammation; provide a clinical biomarker	^205^, ^206^, ^209^
Myocardial infarction	Human	Blood	CD33^high^CD16^low^ neutrophils	An increase in the frequency of hyperactivated	^207^
Heart failure	Mice	Heart	CCR2^+^ neutrophils, CXCR2^+^ neutrophils	Promote inflammation; antigen presentation	^210^
Ischemic stroke	Human	Blood	CXCR4^bright^/CD62L^dim^ neutrophils, CD54^high^CXCR1^low^ neutrophils	Reflect the severity of the disease	^211^
Alzheimer's disease	Human	Blood	CXCR4^high^/CD62L^low^ neutrophils	Reflect the severity of the disease	^212^
Atherosclerosis	Human	Blood	APC‐like neutrophils	Activate the adaptive immune response to promote disease progression	^214^
IBD	Human	Blood, colon tissue	CD177^+^ neutrophils	Play a protective role in IBD through increased bactericidal activity and IL‐22 production	^223^, ^224^
IBD	Mice	Colon tissue	CD17^+^ neutrophils	Promote inflammation	^225^
Ischemia/reperfusion injury (IR)	Mice	Colon tissue	OLFM4^+^ neutrophils	Lead to intestinal damage and mortality after IR injury	^226^
Ulcerative colitis	Human	Colon tissue	CXCR2^+^ neutrophils	Enrich in nonresponders to ustekinumab therapy	^227^
Periodontitis	Human	Blood	CD177^+^ neutrophils	Preferentially recruited to the gingival crevice of periodontitis patients	^230^
Optic nerve and spinal cord injury	Mice	Optic nerve and spinal cord	CD14^+^Ly6G^low^ neutrophils	Neuroprotective role by secreting NGF and IGF‐1	^233^, ^234^
Allergic rhinitis	Mice	Nasal mucosa	Ly‐6G^+^SiglecF^+^ neutrophils	Exhibit an activated phenotype and enhance effector functions	^235^
Erythema nodosum leprosum	Human	Blood	IL‐10R1^+^ neutrophils	Release inflammatory cytokines	^166^
Psoriasis	Human	Skin tissue	CD10^neg^CD16^low^CD11b^low^ neutrophils; CD10^neg^CD16^neg^CD11b^neg^ neutrophils	Induce IL‐17 and IFN‐γ production by T cells	^236^
Wound closure after skin transplantation	Mice	Blood; Skin tissue	CD49d^+^ neutrophils	Play a role in local vessel remodeling during inflammation	^237^
Femur fracture	Rat	Blood	CD11b^high^/CD11a^high^ neutrophils	The number of cells increases significantly in diseases.	^238^

The dual role of neutrophils in diseases ought to get sufficient consideration. On the one hand, as innate immune phagocytes, neutrophils function pivotally in immune defense. On the other hand, specific neutrophil subpopulations would promote disease progression. Extensive and comprehensive research of the functional heterogeneity of neutrophils is envisaged to enable precision intervention on neutrophil subpopulations and achieve precise treatment of inflammation‐related diseases.

### 4.2 Neutrophile heterogeneity in cancer‐related diseases

**FIGURE 5:**
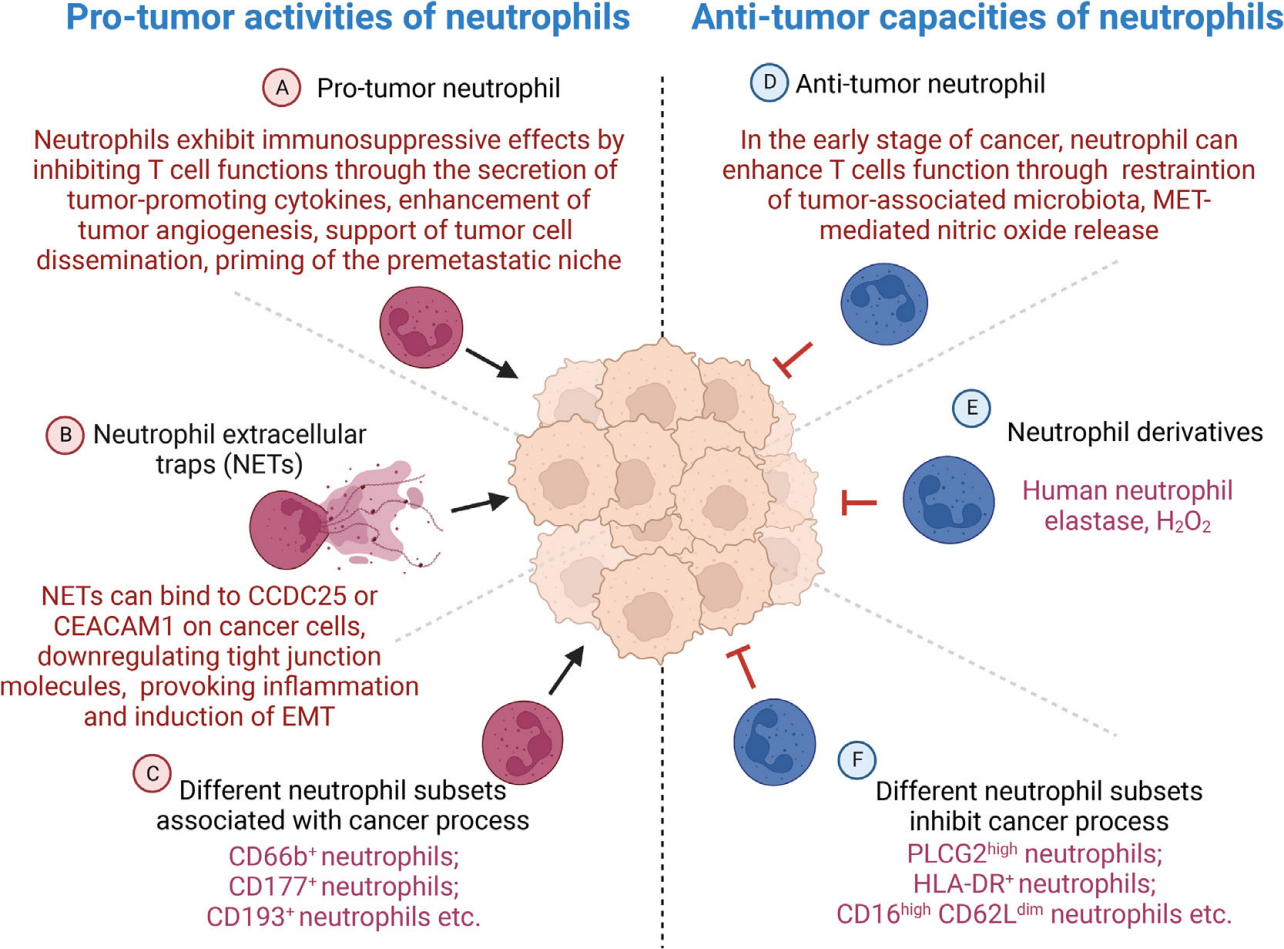
Neutrophils show both protumor activity or antitumor capacities. This figure is created with BioRender.com.

Over years past, accumulating evidence has demonstrated that neutrophils perform dual roles, exhibiting both pro‐ and antitumor activity. Specific mechanisms are associated with tumor cell proliferation, apoptosis, and the immune microenvironment.^240^, ^241^ As study advances, researchers have shown that neutrophils undergo phenotype and functional remodulation under the influence of tumors and their microenvironment, playing a complicated role in tumor development and metastasis.^242^, ^243^, ^244^ Besides protumor activity through protumor neutrophils, NETs, and various subsets associated with cancer process, neutrophils also exert antitumor functions via antitumor neutrophils, other derivatives, and subsets inhibiting cancer process (Figure 5).

#### 4.2.1 Colorectal cancer

Colorectal cancer (CRC) is one of the most prevalent malignancies globally, accounting for 10% of all cancer cases. The incidence of CRC ranked third among malignant tumors, making it the third leading cause of cancer mortality.^245^, ^246^ In recent years, several studies have shown that the double role of neutrophils in inflammation‐related diseases, particularly in the development and metastasis of tumors, has been gradually recognized and has garnered growing attention.^247^, ^248^ Neutrophils and NETs might promote CRC development and metastasis in part by the mediation of tumor metastasis by NETs via binding to CCDC25 or CEACAM1 on cancer cells.^247^, ^249^, ^250^, ^251^, ^252^ Inhibition of NETs release attenuated colitis as well as colitis‐associated tumorigenesis.^253^, ^254^ In addition, neutrophils seemed to be protective against the advancement of CRC. Neutrophil elastase has been proven to be inhibitory effective against 35 kinds of cancer cells, including CRC cells.^255^ Neutrophils restrained tumor‐associated microbiota and decreased colon tumor development and invasion in an inflammatory‐induced CRC mice model, while neutrophil depletion would in contrast made tumors more aggressive.^256^ The role of neutrophils in preventing bacterial invasion may be mediated by IL‐1β.^257^ These findings indicated the intricacy of tumor‐associated neutrophil (TAN) involvement in CRC progression. What is more, neutrophils from CRC patients could also exhibit immunosuppressive effects by inhibiting T cell functions through TGFβ.^258^ CD177^+^ neutrophils and CD66b^+^ neutrophils suppress epithelial cell tumorigenesis in colitis‐associated cancer and became a predictor for a better prognosis in CRC.^259^, ^260^ In the early stages of CRC, the number of CD66b^+^ neutrophils were often associated with a favorable prognostic factor. Specific inductions would stimulate the generation of particular neutrophil subsets.^261^ For instance, the addition of IL‐21 into bone marrow cell culture increased the amount of CD193^+^ neutrophils, which migrated readily into the duodenum.^262^ Moreover, in a genetic CRC model, suppression of neutrophil‐dependent angiogenesis abrogated resistance to anti‐VEGF antibody.^263^ Taken together, these studies suggested that targeting neutrophils is of great significance for CRC treatment.

#### 4.2.2 Hepatocellular carcinoma

The important role of neutrophils in liver cancer has gradually attracted accelerated interest in past few years.^264^, ^265^, ^266^, ^267^ According to the present studies, NETs could promote the development from NASH to hepatocellular carcinoma (HCC).^268^ Besides, NETs promoted HCC metastasis by downregulating tight junction molecules on adjacent endothelial cells or provoking inflammation.^269^, ^270^ Neutrophils, especially type‐2 neutrophils (N2), could mediate the formation of an immunosuppressive microenvironment in liver tissue, and neutrophil clearance in mouse models would impede tumor progression.^271^, ^272^, ^273^ The production of neutrophils for specific functions is usually influenced by a variety of factors. For instance, in several models of liver autoimmunity, pharmacologically induced autoantigen‐specific T regulatory type‐1 (TR1) cells and TR1‐cell‐induced B regulatory (Breg) cells used five immunoregulatory cytokines to coordinately recruit neutrophils into the liver and program their transcriptome to generate regulatory neutrophils.^272^ Additionally, PD‐L1^+^ neutrophils have been shown to promote the growth of tumor cells in tissues from HCC patients by inhibiting the proliferation and activation of T cells.^274^ The generation of PD‐L1^+^ neutrophils was likely attributed to the induction of cancer‐associated fibroblasts through the IL6‐STAT3 pathway.^275^ Another study also confirmed that, in addition to PD‐L1^+^ neutrophils, both CCL4^+^ TANs presented immunosuppressive activity.^276^ Neutrophils also have a significant impact on the treatment of HCC.^277^ Tumor‐derived lactate would inhibit the efficacy of Lenvatinib in HCC by modulating PD‐L1 expression on neutrophils.^278^ The mechanism of action of cabozantinib combined with PD‐1 antibody to enhance antitumor immunity through neutrophil‐based immune responses favored this approach for HCC therapy.^279^ Furthermore, in HCC immunotherapy, PD‐L1^+^ neutrophils would be directly activated by IFN‐γ and subsequently prompting inflammatory response.^280^ Therefore, precise targeting of specific neutrophil subsets might reduce the occurrence of immune‐related adverse events to a significant extent. Although the tumor‐killing effect of neutrophils has been reported in other tumors, it has not been reported in liver cancer, which may be a future research direction.

#### 4.2.3 Gastric cancer

With high morbidity and mortality, gastric cancer (GC) is a ubiquitous malignancy worldwide.^281^, ^282^ Heretofore, surgical treatment remains the first‐line strategy to providing a cure. It is expected that analyzing the molecular events taken place during GC progression would cast new light on GC treatment.^283^ Tumor‐derived exosomes could induce neutrophil activation through the HMGB1/TLR4/NF‐κB signaling pathway during GC progression.^284^ As well, neutrophils were able to promote GC progression by inducing Th17 cell polarization or inhibiting CD8^+^ T cell function.^285^, ^286^ Xia et al.^287^ reported that NETs could promote the metastasis of postoperative abdominal infectious complications in GC patients. Induction of epithelial–mesenchymal transition (EMT) has been verified to be another way for NETs to promote GC metastasis.^288^, ^289^ Five subpopulations of neutrophils were identified in tissues from GC patients. Among them, CXCR4^+^ neutrophils have proangiogenic and proinvasive properties; while PLCG2^high^ neutrophils can inhibit the invasion and migration of tumor cells.^290^ Through single‐cell RNA sequencing, researchers from another team mapped the lymph node metastasis landscape of GC and found that lipocalin 2 (LCN2)^+^ neutrophils served as a promotor in lymph node metastasis. This suggested that neutrophil subsets might drive metastatic in specific tumor sites.^291^ The number of PD‐L1^+^ neutrophils and CD66b^+^ neutrophils are associated with the survival of GC patients.^292^, ^293^, ^294^ Interestingly, the number of FasL^+^PD‐L2^+^ neutrophils was reported to be associated with poor prognosis of GC in another study. These neutrophils owned immunosuppressive functions on tumor‐specific CD8^+^ T cells and promoted the growth and progression of human GC tumors both in vitro and in vivo. Targeting the removal of FasL^+^PD‐L2^+^ neutrophils could hinder GC progression.^295^ The advancement of medical therapy for GC has been quite sluggish thus far. Consequently, the development of targeted drugs for the heterogeneity of neutrophil function is anticipated to yield novel insights for the therapeutic treatment of GC.

#### 4.2.4 Lung cancer

Lung cancer is among the world's most prevalent malignant tumors. The incidence and mortality rate have grown dramatically over the past 50 years, and males were reported to have the highest morbidity and mortality of all malignant tumors.^296^, ^297^ The proportion of neutrophils in lung cancer patients' tissue or peripheral blood can be utilized to predict the prognosis of lung cancer.^298^, ^299^ Studies have demonstrated that neutrophils and NETs they generate play a critical role in fostering lung tumor development and metastasis.^300^, ^301^, ^302^ Among these, senescent TANs (CXCR4^+^CD62L^low^) are more likely to form NETs and facilitate metastasis.^303^ It has been reported by Tyagi et al.^304^ that N2 neutrophils could release LCN2 which would in turn facilitate lung cancer metastasis. The heterogeneity of neutrophils in the peripheral blood of non‐small cell lung cancer patients were greater, and the proportion of LDNs was remarkably upregulated.^305^, ^306^ Faget et al.^307^ reported that Gr1^+^ neutrophils could favor tumor progression by altering angiogenesis, leading to hypoxia, and sustaining Snail expression. Through MET‐mediated nitric oxide release, neutrophils can also abate tumor growth and metastasis.^79^ In recent years, a group of TANs with high expression of SiglecF (sialic acid‐binding immunoglobulin‐like lectin F) has gradually attracted researchers’ attention. SiglecF^high^ neutrophils could selectively upregulate the expression of genes associated with tumor‐processes, including angiogenesis, myeloid cell differentiation and recruitment, extracellular matrix remodeling, suppression of T cell responses, and tumor cell proliferation and growth.^308^ In the early stages of lung cancer, TANs could enhance the T cell activity to display an antitumor effect.^309^ This unique neutrophil population was more mature with a longer lifespan.^98^, ^106^, ^111^ Its function in promoting lung cancer, however, was dependent on the number of osteoblastic cells, suggesting that the function of SiglecF^high^ neutrophils was infected by the ratio of cell types.^308^ Interestingly, another study on mouse lung cancer showed that, similar to SiglecF^high^ neutrophils, neutrophils with Glut1 high expression could live longer in the tumor microenvironment, and could promote the high expression of SiglecF and MMP9, facilitating tumor progression.^310^, ^311^ However, whether Glut1^high^ neutrophils and SiglecF^high^ neutrophils are the same population of neutrophils remains unclear. Another research team identified a group of ISG neutrophils in mouse lung tissue, and the number of ISG neutrophils was significantly reduced in tumor tissue.^312^ A subset of tumor‐associated HLA‐DR^+^ neutrophils was also identified in early‐stage human lung cancer tumor tissue, which can cross‐present antigens to CD8^+^ T cells, triggering antitumor T cell immune responses.^313^ A group of CD66b^+^/CD10^low^/CXCR4^+^/PDL1^inter^ neutrophils were identified in the peripheral blood of advanced lung cancer patients, the existence of which was limited in the advanced stage of lung cancer.^90^ At present era, neutrophils have promising potential as effector cells in the treatment of lung cancer. By modifying neutrophil infiltration, for instance, the CXCR2 selective inhibitor SB225002 can boost the therapeutic effect of cisplatin in the treatment of lung cancer.^314^ To sum up, neutrophil heterogeneity is extremely important in the prognosis, progression, and treatment of lung cancer. Unfortunately, studies of neutrophil heterogeneity in specific types of lung cancer are limited. For example, it has been reported that neutrophil to lymphocyte ratio predicts the prognosis of extensive small‐cell lung cancer.^315^ However, there are few studies on neutrophil heterogeneity in small cell lung cancer.

#### 4.2.5 Breast cancer

Breast cancer is the most common malignancy in women and one of the three most common cancers worldwide, along with lung and colon cancer.^316^ Numerous studies have demonstrated that neutrophils could promote the growth and metastasis of breast cancer via the secretion of cytokines and other pathways,^317^, ^318^, ^319^ as well as facilitate breast cancer metastasis by altering other cell functions, such as inhibiting the antimetastasis function of NK cells or mediating the immunosuppressive microenvironment.^320^, ^321^ Tumor‐derived factors such as G‐CSF and stem cell factor could in turn responsible for activity of immunosuppressive neutrophils, thereby contributing to the advancement of breast cancer.^109^, ^322^ Interestingly, during the premetastatic stage of breast cancer, tumor‐entrained neutrophils in the lung aggregated and, by generating H_2_O_2_, might limit metastatic seeding in the lungs.^80^ This partly explains the functional differences of neutrophils at the primary site and the metastatic site. Neutrophils could also play a protumor role by affecting the lipid metabolism of tumor cells. In the context of a low glucose supply in the tumor microenvironment of 4T1 tumor‐bearing mice, neutrophils would engage in fatty acid oxidation, supporting ROS production and amplifying T cell suppression.^109^ In addition, a separate study reported that lipids stored within lung neutrophils can be transported to metastatic tumor cells through a macropinocytosis–lysosome pathway, endowing tumor cells with augmented survival and proliferative capacities.^301^ Already published is the discovery that neutrophils could exert a mode of destruction of breast cancer cells has already been reported, and the specific mode was related to the regulation function of neutrophils toward CD47‐SIRPα checkpoint through the trogoptosis mechanism.^323^, ^324^ It was identified by Veillette et al.^324^ that C5aR1^+^ neutrophils would predict worse prognosis of BC patients, which could mechanistically be explained by the ability of C5aR1^+^ neutrophils in inducing breast cancer glycolysis via increasing ERK1/2‐WTAP‐dependent m6A methylation of ENO1.^325^ Moreover, Coffelt et al.^326^ reported that in mammary tumor‐bearing *K14^cre^;Cdh1^F/F^;Trp53^F/F^* (KEP) mice, the tumor microenvironment favors a unique population of metastasis‐inducing neutrophils with upregulation of genes including *cKit*, *Nos2*, *Prok2*, *S100a8*, and *S100a9*. Using a panel of 16 distinct genetically engineered mouse models for breast cancer, another analysis also identified the significant enrichment of cKIT^+^ neutrophils was more likely to potentiates metastatic progression.^327^ In previous studies, neutrophil Cathepsin G was found to interact with RAGE (receptor for advanced glycation end products), displaying its antitumor activity.^328^ By releasing metastasis‐promoting factors including forming NETs, CXCR4^hi^CD62L^lo^ aged neutrophils could enhance metastasis of breast cancer cells to the liver or lung.^303^, ^329^ In summary, neutrophils play a dual role in breast cancer progression. The distinction of which may be attributed to the various phases of the disease and the heterogeneity of neutrophils. which merit additional investigation.

#### 4.2.6 Pancreatic cancer

Pancreatic cancer is one of the most aggressive tumors of the digestive system. The mortality rate is rising annually due to the its stealthy onset, fast development, and ineffective treatment.^330^, ^331^ Multiple research teams have proven that neutrophils and NETs may greatly enhance the growth and metastasis of pancreatic cancer, and that inhibiting NETs can somewhat delay pancreatic cancer metastasis.^332^, ^333^, ^334^ In an orthotopic pancreatic cancer mouse model, the presence of neutrophils was shown to be associated with functional suppression of the CD8^+^ T cells.^334^ In the pancreatic tumor microenvironment, TAN subsets with various functions exist, among which BHLHE40^+^ neutrophils have protumor and immunosuppression functions.^335^ A recent research revealed that P2RX1^neg^ neutrophils were highly immunosuppressive, hence provoking pancreatic cancer liver metastasis.^335^ CD13^hi^ neutrophil‐like myeloid‐derived suppressor cells exert immune suppression in pancreatic ductal adenocarcinoma by influencing the expression of Arginase 1.^336^ Targeting neutrophils has also showed encouraging effectiveness in the treatment of pancreatic cancer.^337^ Lorlatinib attenuates pancreatic cancer growth by suppressing TAN amount and functions.^338^ The therapeutic effect of nivolumab in pancreatic cancer was in association with CD11b^+^ neutrophil degranulation.^339^ Steele et al.^340^ revealed that mediating the expression of CXCR2 on neutrophils could improve the efficacy of PD‐1 antibodies in a pancreatic cancer mouse model. This may be due to the fact that high expression of CXCR2 on neutrophils would mediate immunosuppressive microenvironment, effecting T cell entry.^340^, ^341^ Targeting tumor‐associated CXCR2^+^ neutrophils and CCR2^+^ macrophages was reported to improve chemotherapeutic responses in pancreatic ductal adenocarcinoma.^342^ In a nutshell, a series of studies have shown that it is necessary to investigate neutrophil heterogeneity in the context of pancreatic cancer treatment.^266^, ^267^, ^268^, ^269^, ^270^, ^271^, ^272^, ^273^, ^274^, ^275^, ^276^, ^277^, ^278^, ^279^, ^280^, ^281^, ^282^, ^283^, ^284^, ^285^, ^286^, ^287^, ^288^, ^289^, ^290^, ^291^, ^292^, ^293^, ^294^, ^295^, ^296^, ^297^, ^298^, ^299^, ^300^, ^301^, ^302^, ^303^, ^304^, ^305^, ^306^, ^307^, ^308^, ^309^, ^310^, ^311^, ^312^, ^313^, ^314^, ^315^, ^316^, ^317^, ^318^, ^319^, ^320^, ^321^, ^322^, ^323^, ^324^, ^325^, ^326^, ^327^, ^328^, ^329^, ^330^, ^331^, ^332^, ^333^, ^334^, ^335^, ^336^, ^337^, ^338^, ^339^, ^340^, ^341^, ^342^, ^343^ For subsequent investigation, the inductive factors of the emergence of diverse neutrophil subsets may be probed, enabling a brand‐new viewpoint for the treatment of pancreatic cancer.

#### 4.2.7 Other cancers

**FIGURE 6:**
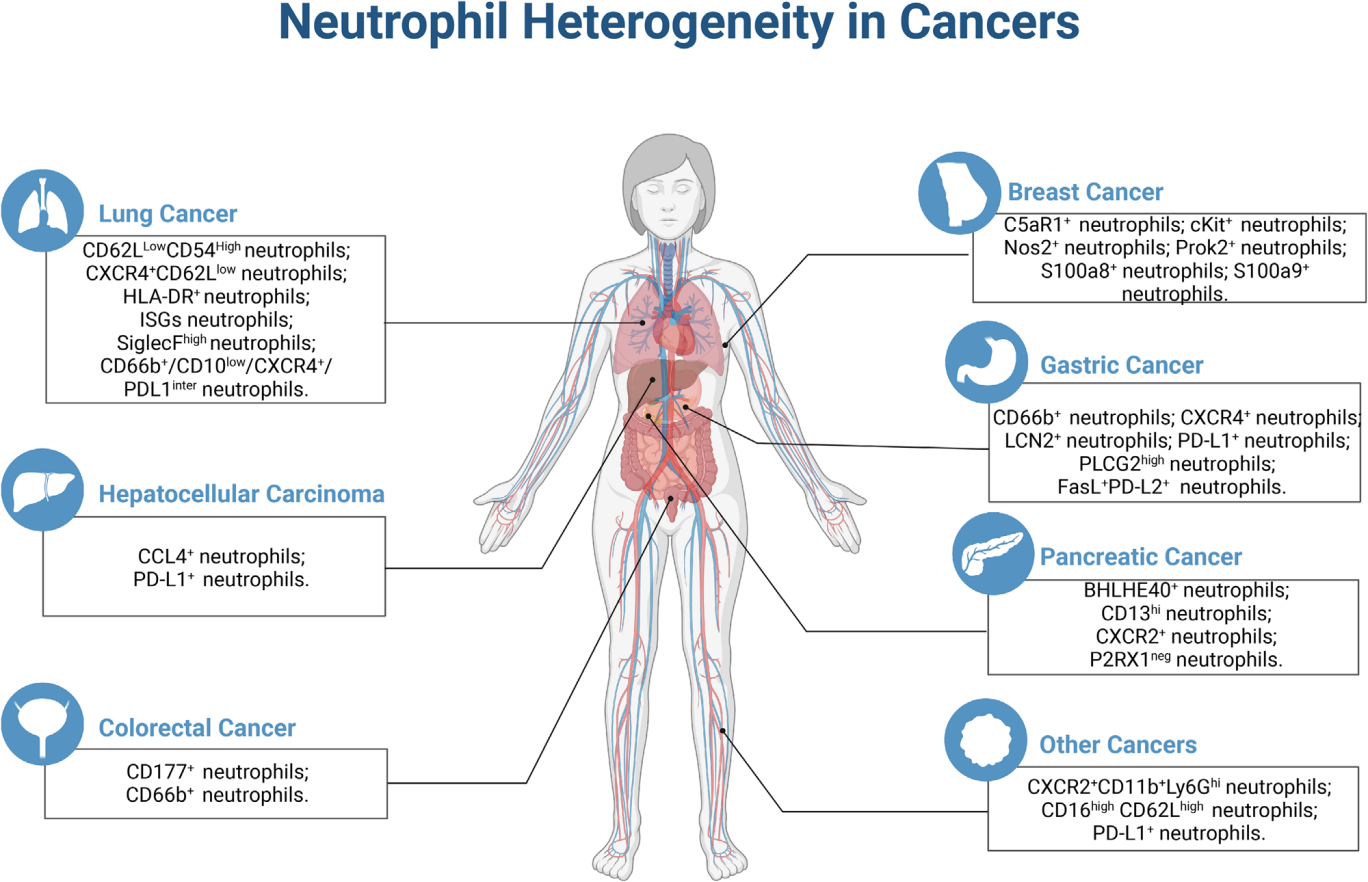
Neutrophil heterogeneity in cancers. This figure is created with BioRender.com.

Researchers are also intrigued by the role of neutrophil heterogeneity in other cancers. Neutrophils could regulate the function of CD47‐SIRPα checkpoint through trogoptosis, presenting tumor‐suppressive property in acute promyelocytic leukemia.^344^ Meanwhile, combination of SIRPα antibody with CD70 antibody could, to some extent, enhance the antitumor effect of neutrophils.^345^ Whether this process is dependent on neutrophils remain obscure. In addition, neutrophils also presented tumoricidal effect in neuroblastoma and endometrial cancer, the mechanism of which might be correlated with the induction of MMP9 expression and ROS generation.^346^, ^347^, ^348^ The ROS‐dependent antitumor effect of neutrophils has also been demonstrated in a mesothelioma mouse model.^349^ However, another study showed that CXCR2^+^CD11b^+^Ly6G^hi^ myeloid‐derived suppressor cells (mainly expressing neutrophil marker genes) affected the efficacy of PD‐1 antibodies in rhabdomyosarcoma mice.^350^ Three neutrophil subsets were identified in head and neck squamous cell carcinoma (HNSCC) patients: CD16^dim^ CD62L^high^, CD16^high^ CD62L^high^ and CD16^high^ CD62L^dim^ neutrophils. Among them, CD16^high^ CD62L^dim^ neutrophils could inhibit the migration and proliferation of HNSCC cells, as well as inducing cell apoptosis.^351^ Another study showed that immunosuppressive neutrophil subsets, such as CD16^high^ CD62L^high^ neutrophils, might be induced by tumor‐produced TGF‐β/IL‐10.^352^ Six phenotypically distinct human blood neutrophil populations were identified. The proportion and function of neutrophil subsets were shown to be correlated with melanoma stage, and their changes would favor the differentiation of different melanoma stages.^353^, ^354^ The profound study of neutrophils has also given hope for the treatment of melanoma. A recent study showed that neutrophils, especially TAN1, were isolated from mice underwent innate immune training by exposure to β‐glucan hold a stronger antitumor effect.^355^ The activity of alemtuzumab against B‐cell chronic lymphocytic leukemia was reported to be dependent on neutrophil‐mediated cytotoxicity.^356^ Combination of CTLA‐4 and PD‐L1 blockade in patients with chemotherapy‐naive metastatic castration‐resistant prostate cancer was shown to be associated with the modulation of neutrophil subsets in the bone microenvironment.^357^ Collectively, neutrophil heterogeneity has been observed in a variety of cancers (Figure 6). The subsets of neutrophils, however, seem to vary from tissue to tissue, requiring researchers to follow up with investigations in specific tumors (Table 2).

**TABLE 2:** Neutrophil heterogeneity in cancer‐related diseases.

Diseases	Species	Samples	Neutrophil heterogeneity	Functions	References
Colorectal cancer	Human	Colon tissues	CD177^+^ neutrophils	Suppress epithelial cell tumorigenesis	^262^
Colorectal cancer	Human	Colon tissues	CD66b^+^ neutrophils	A favorable prognostic factor in early stages of colon cancer	^263^
Hepatocellular carcinoma	Mice	Liver tissues	N2 neutrophils	Immunosuppressive function	^275^
Hepatocellular carcinoma	Human, mice	Liver tissues	PD‐L1^+^ neutrophils	Immunosuppressive function	^277^, ^278^
Hepatocellular carcinoma	Human, mice	Liver tissues	CCL4^+^ neutrophils	Recruit macrophages	^279^
Hepatocellular carcinoma	Mice	Liver tissues	PD‐L1^+^ neutrophils	Promote inflammatory response	^283^
Gastric cancer	Human	Gastric tissues	CXCR4^+^ neutrophils; PLCG2^high^ neutrophils	Proangiogenesis; inhibit tumor cell invasion and migration	^293^
Gastric cancer	Human	Gastric tissues; metastatic lymph nodes	LCN2^+^ neutrophils	Contribute to lymph node metastasis	^294^
Gastric cancer	Human	Gastric tissues	PD‐L1^+^ neutrophils	Associated with disease progression and reduced GC patient survival	^295^
Gastric cancer	Human	Gastric tissues	CD66b^+^ neutrophils	Predict the poor disease special survival	^296^, ^297^
Gastric cancer	Human	Gastric tissues	FasL^+^PD‐L2^+^ neutrophils	Immunosuppressive function	^298^
Breast cancer lung metastasis	Mice; Human	Lung tissues	CXCR4^+^CD62L^low^ neutrophils	Contribute to breast cancer lung and liver metastasis	^306^, ^331^
Breast cancer lung metastasis	Mice; Human	Lung tissues	N2 neutrophils	Contribute to breast cancer lung metastasis	^307^
Lung cancer	Human	Blood	Low‐density neutrophils	The number of cells increases significantly in patients	^79^
Lung cancer	Mice	Lung tissues	Gr1^+^ neutrophils	Immunosuppressive function	^309^
Lung cancer	Human	Lung tissues	CD62L^Low^CD54^High^ neutrophils	Release inflammatory cytokines	^312^
Lung cancer	Human	Lung tissues	SiglecF^high^ neutrophils	Promote tumor growth by inducing Vegfa, Ctsb and Tgfb1	^311^
Lung cancer	Human	Lung tissues	ISG neutrophils	The number of cells decreases significantly in patients.	^317^
Lung cancer	Human	Blood	HLA‐DR^+^ neutrophils	Enhance immune response	^318^
Lung cancer	Human	Blood	CD66b^+^/CD10^low^/CXCR4^+^/PDL1^inter^ neutrophils	Relate to advanced lung cancer diagnosis	^90^
Breast cancer	Human	Tumor tissues	C5aR1^+^ neutrophils	Induce breast cancer glycolysis and promote tumor growth	^328^
Breast cancer	Human	Tumor tissues	Neutrophils (cKit^+^, Nos2^+^, Prok2^+^, S100a8^+^, S100a9^+^)	Promote tumor metastasis	^329^
Breast cancer	Mice	Tumor tissues	cKit^+^ neutrophils	Promote tumor metastasis	^330^
Pancreatic ductal adenocarcinoma	Human	Blood, tumor tissues	BHLHE40^+^ neutrophils	Immunosuppressive function	^338^
Pancreatic ductal adenocarcinoma	Human	Tumor tissues	P2RX1^neg^ neutrophils	Immunosuppressive function	^339^
Pancreatic ductal adenocarcinoma	Human	Tumor tissues	CD13^hi^ neutrophils	Immunosuppressive function	^340^
Pancreatic ductal adenocarcinoma	Human	Tumor tissues	CXCR2^+^ neutrophils	Immunosuppressive function	^345^, ^346^, ^347^
Rhabdomyosarcoma	Mice	Tumor tissues	CXCR2^+^CD11b^+^Ly6G^hi^ neutrophils	Immunosuppressive function	^354^
Neck squamous cell carcinoma	Human	Blood	CD16^high^ CD62L^high^ neutrophils	Correlate with increased survival rate	^355^
Melanoma	Human	Blood	PD‐L1^+^ neutrophils	Correlate with decreased survival rate	^358^

A comprehensive study of neutrophil heterogeneity would favor us in acquiring a deeper understanding of disease mechanisms and may yield novel insights into cancer treatment.

## 5 OUTLOOK

In this review, we have provided a comprehensive summary of the significance of neutrophil heterogeneity in inflammatory and tumor‐associated diseases. The presence of neutrophils with different phenotypes in blood, peripheral blood, or tissue has been confirmed by conventional or high‐resolution methods. However, two major challenges regarding neutrophil heterogeneity exist currently. One is to understand the underlying causes of neutrophil heterogeneity, while the other is how neutrophil subsets are defined. There are two potential mechanisms for neutrophil heterogeneity. The first is the intrinsic heterogeneity of neutrophils present in the bone marrow and blood. The second involves exposure to extrinsic factors, either locally or systemically, that modify neutrophil properties.^83^ It is noteworthy that the factors inducing neutrophil heterogeneity vary between different diseases, as previously discussed in the context of inflammatory diseases and tumors. It remains difficult to ascertain whether the occurrence of neutrophil heterogeneity is due to a single signal or a combination of multiple factors. This requires more detailed exploration. Additionally, the correlation between neutrophil functions and the phenotypes identified by surface markers is an important issue that warrants discussion. For example, neutrophils with immunosuppressive function and high expression of PD‐L1 would be defined as PD‐L1^+^ neutrophils.^276^ Although some high‐resolution assays such as RNA‐seq have helped in providing functional information of neutrophil subsets and specific expression of maker genes, the relationship between the neutrophil functions and surface makers is still unclear. This represents an important direction of future research.

Despite the fact that extensive evidence has supported the importance of neutrophils as effector cells for disease treatment, many uncertainties remain to be addressed. Currently, studies of neutrophil heterogeneity have been concentrated on the association level. In the upcoming investigation, functions of multiple neutrophil subpopulations should be evaluated based on various transgenic mice. Simultaneously, comprehensive and accurate definition of neutrophil subpopulations along with factors promoting their formation deserve further investigation. Neutrophils exhibit species specificity, and human and mouse neutrophils might function differently in some respects.^312^, ^358^ Cui et al.^255^ reported that human neutrophil elastase selectively killed tumor cells, whereas similar effects did not occur with mouse neutrophil elastase. This indicated that our research of neutrophil heterogeneity in mice did not immediately correlate with clinical cases. In addition, the role of neutrophils also varies widely across different tumors. Neutrophils with high MET expression had a great antitumor effect in the lung cancer model. In the melanoma model, however, the expression of MET would greatly limit the effect of immunotherapy.^79^, ^359^ Neutrophils function based on the tissue type, the tissue microenvironment, and the stage of disease. Even within the same disease situation, it is possible for neutrophils to perform conflict functions. To be specific, neutrophils showed an antitumor effect by inducing ferroptosis in glioblastoma through MPO; however, the presence of neutrophils in glioblastoma was often seen as a prediction of poor prognosis.^360^ The function of neutrophil subsets in peripheral blood from volunteers of different sexes also differs.^361^ Differences in neutrophil heterogeneity across species, genders, and disease types have brought great challenges to our study of neutrophil heterogeneity, and will continue to be the focus and difficulty of future research.

From a therapeutic standpoint, investigations targeting specific neutrophil subpopulations are being refined. It is of great significance to develop drugs that target particular neutrophil subpopulation for treatment of inflammation and cancer‐related diseases. Though, approaches that would target particular neutrophil subpopulation remain limited and warrant additional investigation. Presently, some neutrophil‐targeted nanoparticles and nanoformulations targeting specific neutrophil subpopulations have shown favorable alleviative effects.^362^, ^363^ This may be due to innate ability possessed by cells to sense, integrate and respond to the dynamic environment within the body, rendering them suitable as carriers of therapeutic drugs. In recent years, the design of drug delivery system regarding neutrophils has become a prominent research direction in the field of drug delivery.^364^, ^365^ There are two types of drug delivery systems: neutrophils as carriers and neutrophil‐membrane‐derived nanovesicles.^364^ Several studies have demonstrated that neutrophil‐based drug delivery systems can improve current therapies for inflammatory disorders and cancers. To be specific, Zhang and coworkers^366^ prepared a tumor‐penetrating neotype neutrophil cytopharmaceutical (NEs@STING‐Mal‐NP) that conjugated liposomal STING agonists on the surface of neutrophils, inheriting the merits of neutrophils such as proactive tumor vascular extravasation and tissue penetration, significantly boosting the tumor penetration of STING agonists. However, one challenge with neutrophil‐based drug delivery systems is determining the optimal time to deliver nanoparticles for better targeting of infiltrated neutrophils. After all, the time course of neutrophil infiltration is heavily reliant on the pathogenesis of the disease and its stages. The use of effector neutrophil subsets in specific disease context to design drug‐delivery systems may offer a novel approach.

Overall, we have provided information on the fundamental function of neutrophils, the double‐edged role of neutrophils in diseases, and the important function of neutrophilic heterogeneity in inflammatory diseases and tumors. Additionally, potential future research directions for neutrophils have also been discussed. We hope that this review would provide a comprehensive understanding of neutrophils.

## AUTHOR CONTRIBUTION

Qu Jiao contributed to the conception and acquisition. Wencheng Zhou and Xinran Cao drafted the manuscript. Qu Jiao and Xinran Cao critically revised the manuscript. Qiang Xu and Yang Sun gave final approval. All authors agree to be accountable for all aspects of work ensuring integrity and accuracy. All authors have read and approved the final manuscript.

## CONFLICT OF INTEREST STATEMENT

The authors have no conflicts of interest to declare.

## ETHICS STATEMENT

Not applicable.

## Data Availability

Not applicable.
